# Interplay between Human Cytomegalovirus and Intrinsic/Innate Host Responses: A Complex Bidirectional Relationship

**DOI:** 10.1155/2012/607276

**Published:** 2012-05-31

**Authors:** Giada Rossini, Cristina Cerboni, Angela Santoni, Maria Paola Landini, Santo Landolfo, Deborah Gatti, Giorgio Gribaudo, Stefania Varani

**Affiliations:** ^1^Section of Microbiology, Department of Hematology and Oncology “L. & A. Seragnoli”, University of Bologna, 40138 Bologna, Italy; ^2^Department of Molecular Medicine, Istituto Pasteur-Fondazione Cenci Bolognetti, Sapienza University of Rome, Rome, Italy; ^3^Department of Public Health and Microbiology, University of Turin, Turin, Italy

## Abstract

The interaction between human cytomegalovirus (HCMV) and its host is a complex process that begins with viral attachment and entry into host cells, culminating in the development of a specific adaptive response that clears the acute infection but fails to eradicate HCMV. We review the viral and cellular partners that mediate early host responses to HCMV with regard to the interaction between structural components of virions (viral glycoproteins) and cellular receptors (attachment/entry receptors, toll-like receptors, and other nucleic acid sensors) or intrinsic factors (PML, hDaxx, Sp100, viperin, interferon inducible protein 16), the reactions of innate immune cells (antigen presenting cells and natural killer cells), the numerous mechanisms of viral immunoevasion, and the potential exploitation of events that are associated with early phases of virus-host interplay as a therapeutic strategy.

## 1. Introduction

Human cytomegalovirus (HCMV) is a ubiquitous, highly specific herpesvirus. As the other herpesviruses, after an initial primary infection HCMV establishes latency for the life of the host with periodic and spontaneous reactivation. In immunocompetent subjects, primary HCMV infection is usually asymptomatic but occasionally gives rise to a self-limited mononucleosis-like syndrome. In immunocompromised patients, HCMV is one of the most common opportunistic pathogens and causes different clinical syndromes, whose severity parallels the degree of the immunosuppression [1]; in these patients HCMV infection causes both direct effects, reflecting cell destruction by the virus, and indirect effects, such as acute and chronic rejection, cardiovascular disease, and HCMV-associated opportunistic infections [2]. During the acute phase of infection, HCMV can infect a remarkably broad cell range within its host, including endothelial cells, epithelial cells, smooth muscle cells, fibroblasts, neuronal cells, hepatocytes, trophoblasts, monocytes/macrophages (M*φ*s), and dendritic cells (DCs) [3].

HCMV induces many hallmarks of innate immune responses, such as the production of inflammatory cytokines and activation of the interferon (IFN) pathway in both immunocompetent and immunocompromised patients. This induction is rapid and does not require transcriptionally active viral particles [4]. The ability of the soluble forms of envelope glycoproteins B (gB) and H (gH) to effect a similar pattern of cellular responses suggests that their interactions with host cell components, such as integrin heterodimers, toll-like receptors, and entry receptors, are sensed by host cells, leading to early signaling and transcriptional events in infected cells and activating innate immune responses before the outset of viral replication [4–6].

Proper activation of innate immunity appears to be crucial to efficiently combat infections; in addition to the release of primary IFNs, professional antigen-presenting cells (APCs) are activated and natural killer (NK) cells are recruited and stimulated, triggering APCs and T cells. Further, unlike the innate and adaptive components of the immune system that require pathogen-induced signaling cascades for activation, intrinsic immune mechanisms are significant, forming an antiviral frontline defense that is mediated by cellular proteins, called restriction factors, that are constitutively expressed and active, even before a pathogen enters a cell [7–9]. Notably, interplay exists between innate and intrinsic immune mechanisms, wherein several restriction factors are upregulated by IFN, enhancing their antiviral activity [10, 11].

This paper describes the viral and cellular partners that mediate early host responses to HCMV with regard to the interaction between structural components of virions and cellular receptors and intrinsic factors, the reactions of innate immune cells, the mechanisms of viral immunoevasion, and the potential exploitation of events that are associated with these early phases of virus-host interplay as a therapeutic strategy.

## 2. Binding and Activation: Function of Receptors in Early Stages of HCMV Infection

Several receptors, including epidermal growth factor receptor (EGFR) [12, 13], platelet-derived growth factor receptor (PDGFR)-*α* [14], and integrins [15, 16], mediate HCMV attachment and entry. Virus-receptor interactions appear to be cell-type specific. For example, in the interaction between HCMV and monocyte-derived dendritic cells (Mo-DCs), viral envelope glycoprotein gB binds to the DC membrane protein DC-SIGN [17]. Polymorphisms in the promoter of *DC-SIGN* that enhance its expression on the surface of Mo-DCs are linked to higher levels of HCMV infection *in vitro* and *in vivo* [18], implicating DC-SIGN in viral entry into DC-SIGN-positive immune cells.

In addition to its binding to receptors, facilitating its entry, the virus is sensed by pattern recognition receptors (PRRs), such as toll-like receptors (TLRs), which initiate immune responses by recognizing pathogen-associated molecular patterns (PAMPs). TLR activation is followed by inflammatory cytokine secretion, upregulation of costimulatory molecules on APCs, and, in most cases, type I IFN production [19].

The initial evidence that HCMV activates innate immunity in a TLR-dependent manner was obtained with TLR2; stimulation of TLR2 by HCMV is replication independent and results in the activation of NF-*κ*B and the release of inflammatory cytokines [20] without affecting the IFN pathway [21]. The envelope glycoproteins gB and gH also interact with TLR2, and neutralizing antibodies against TLR2, gB, and gH inhibit inflammatory cytokine responses to HCMV infection in permissive human fibroblasts [22]. Further, HCMV fusion inhibitors block virus-induced IFN signaling but not inflammatory cytokine secretion, suggesting that the latter is effected by surface sensing by TLR2 and does not require viral entry [21]. These findings indicate that HCMV-induced activation of cell surface TLR2 occurs at the earliest stages of infection; that is, the recognition and binding of envelope glycoproteins. 

In addition to the *in vitro* findings, there is clinical evidence that implicates TLR2 in the pathogenesis of HCMV infection; liver transplant recipients who carry the homozygous Arg753Gln mutation of TLR2 have a higher incidence of HCMV-related disease that is associated with increased levels of HCMV DNA in the peripheral blood [23]. This clinical finding is explained by *in vitro* data that cells with the Arg753Gln mutation in TLR2 fail to recognize HCMV gB. Thus, impaired innate viral recognition might impede the development of a robust antiviral immune response, resulting in symptomatic disease in immunocompromised transplant recipients [24]. Chan and Guilbert have also demonstrated the significance of TRL2 in the immunopathogenesis of HCMV, reporting that UV-inactivated virions stimulate apoptosis in syncytiotrophoblast-like cells in a TLR2-dependent manner, likely contributing to chronic villitis and disruption of syncytiotrophoblasts, which often develop in placentas on delivery of newborns with congenital HCMV [25].

Intracellular TLRs, including TLR3, TLR7, TLR8, and TLR9, detect nucleic acids and are primarily involved in viral detection; TLR3, 7, and 9 recognize microbial nucleic acids in endolysosomes and trigger innate and downstream adaptive immune responses [26]. Endosomal TLR3 and TLR9 are essential components in the innate response to murine CMV (MCMV) in DCs and M*φ*s, and TLR9 is critical for NK cell activation and control of MCMV infection [27–29]. TLR9 also functions in the early responses to HCMV in humans; HCMV induces IFN-*α* secretion from human plasmacytoid DCs (PDCs) by engaging the TLR7 and/or TLR9 pathways *in vitro *[30] and upregulates TLR9 expression in human PDCs [30] and fibroblasts [31].

Notably, the stimulation of TLR9 by its ligand, CpG-B, when added after viral entry, enhances HCMV infection in fibroblasts by an unknown mechanism, suggesting that the virus exploits TLR9 signaling to further its replication during infection of stromal cells. Moreover, the presence of T-1237C polymorphism that alters TLR9 promoter activity [32] correlates with symptomatic HCMV infection in stem cell transplants [33], implicating the TLR9 pathway in the recognition of and response to HCMV.

HCMV infection in fibroblasts is also influenced by the TLR3 and TLR4 pathways; stimulation of fibroblasts with TLR3 and TLR4 ligands inhibits viral replication through an IFN-*β*-dependent mechanism [31, 34]. Nevertheless, TLR3 has no function in the innate/early phases of the cellular response to HCMV in human Mo-DCs, as recently demonstrated by experiments in which TLR3 was silenced before HCMV infection [35]. HCMV also triggers TLR-independent DNA sensing mechanisms [36], as evidenced by findings that the DNA sensor ZBPI/DNA-dependent activator of IFN-regulatory factors (DAI) activates IFN regulatory factor (IRF) 3 and upregulates type I IFN on HCMV infection [37]. Further, HCMV modulates the activity of other innate immunity receptors that induce type I IFN secretion, such as retinoic acid-inducible gene I (RIG-I-) like helicases (RLHs); RIG-I is upregulated quickly in the early phase of HCMV infection in fibroblasts [38].

**Figure 1:**
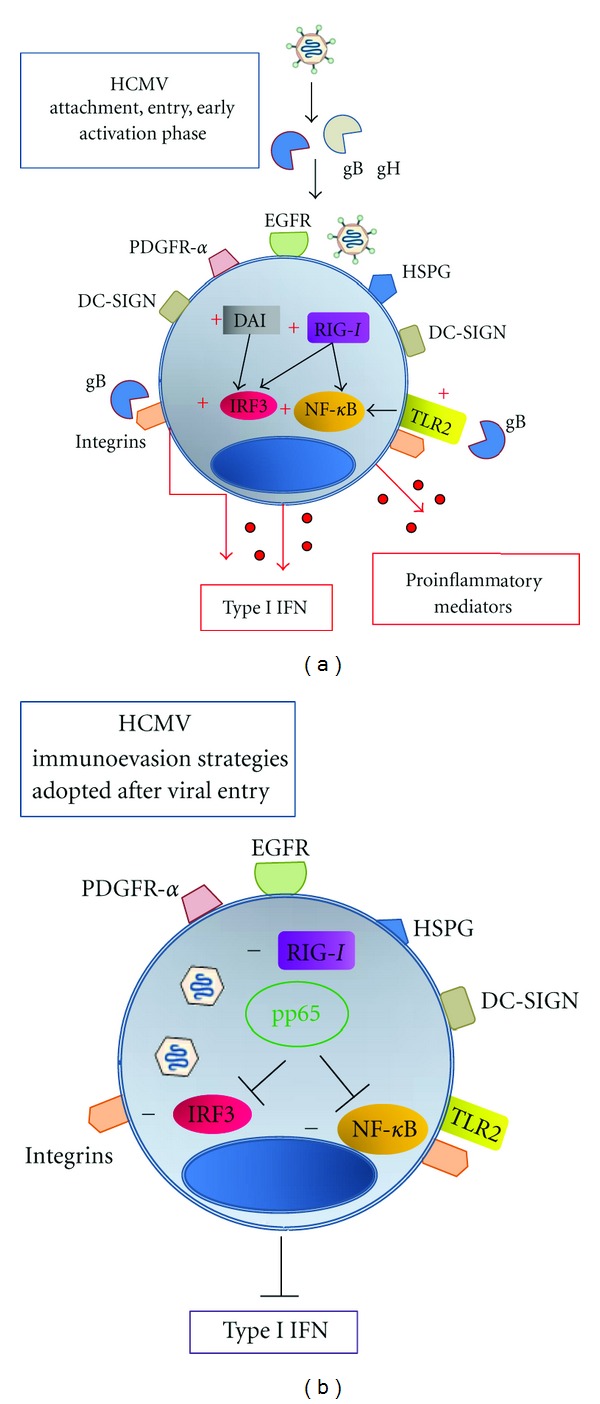
Activation and viral-induced modulation of early phases, HCMV attachment, entry, and intracellular phases of the viral cycle. (a) The binding of viral glycoprotein B (gB) induces the release of type I interferons (IFN)* via* IFN regulatory factor (IRF) 3, whereas contact between viral glycoproteins gB and gH and toll-like receptor (TLR)2 induces the activation of NF-*κ*B and the release of proinflammatory cytokines. Expression of the intracellular receptor retinoic acid-inducible gene I (RIG-I) is also upregulated in the early phases, the DNA sensor DNA-dependent activator of IFN-regulatory factors (DAI) is activated, triggering IRF-3 activation and type I IFN production. (b) After viral entry, HCMV immunoevasion strategies are activated. Virion-associated and newly produced pp65 prevents IRF3 activation and subsequently impairs the production of type I IFN. Viral pp65 also inhibits NF-*κ*B activation. RIG-I is downmodulated by an unknown mechanism, likely contributing to reduced IFN production. +; upregulation or activation, −; downmodulation or inhibition.

Other HCMV attachment/entry receptors might mediate the development of innate responses. Because they associate with TLRs [39] and HCMV glycoproteins [15, 40, 41], surface integrins have been proposed to facilitate the interactions of gB and gH with TLR2 [22, 42]. However, the ligation of gB to *β*_1_ integrin stimulates IFN signaling but not NF-*κ*B-mediated inflammatory signalling [21], suggesting that this interaction induces a TLR-independent antiviral state before viral entry. The activation of innate mechanisms following HCMV attachment and entry and virus-induced modulation of host responses is depicted in Figure 1.

HCMV infects a variety of nonimmune cells *in vivo*, including fibroblasts, endothelial cells, epithelial cells, smooth muscle cells, and stromal cells; each of which expresses a unique subset of TLRs and other innate receptors, allowing them to respond specifically to HCMV infection and contribute to early antiviral defense. The activation of immune receptors on HCMV infection has significant function in fibroblasts [21, 22, 31]. HCMV-induced activation of innate receptors in other nonimmune cells might also be critical, an area that merits further study.

### 2.1. Viral Escape Starts at Very Early Phases

After viral entry, HCMV immunoevasion strategies are activated. The expression of HCMV pp65/UL83 blocks IRF3 signaling, which lies downstream of the RIG-I, DAI, and TLR3 pathways; pp65-mediated impairment of IRF3 signalling occurs by reducing IRF phosphorylation status and by inhibiting its nuclear accumulation [43]. pp65 also blocks IRF1 and NF-*κ*B activation by an unknown mechanism [44], suggesting that HCMV counteracts the activation of the IFN and proinflammatory pathways at several steps. Further, RIG-I is downmodulated by an unknown mechanism starting at 48-hour postinfection [38], likely contributing to reduced IFN production.

## 3. Function of IFN Inducible Restriction Factors in Antiviral Defense

Intrinsic immune mechanisms were discovered as being active against retroviruses and involving the APOBEC3 class of cytidine deaminases, a large family of proteins that are collectively termed the TRIM family, and tetherin, an IFN-inducible protein whose expression blocks the release of HIV-1. Increasing evidence, however, suggests that such mechanisms also counter other viruses [45, 46]. Moreover, four proteins, promyelocytic leukemia protein (PML) [47], hDaxx [48], Sp100 [49], and viperin [50], have been identified as restriction factors that mediate intrinsic immunity against HCMV infection.

PML and hDaxx are components of subnuclear structures called nuclear domain 10 (ND10) or nuclear bodies (NBs). Direct evidence for their antiviral function comes from studies of cells that lack ND10. Primary human fibroblasts from which PML was depleted by small interfering RNA (siRNA) significantly increased the plaque-forming efficiency of HCMV due to enhanced immediate early (IE) expression. hDaxx represses HCMV IE expression and replication through histone deacetylases (HDACs), inducing transcriptionally inactive chromatin around the major IE promoter (MIEP) [51]. These findings demonstrate that the ND10 proteins PML and hDaxx are restriction factors that silence HCMV IE expression, thus controlling viral replication.

Viperin is an IFN-inducible iron-sulfur (Fe-S) cluster-binding antiviral protein that is induced in various cell types by type I, II, and III IFNs and on infection by many viruses, including HCMV. Ectopic expression of viperin in fibroblasts has no effect on the expression of HCMV IE1 or IE2, whereas the synthesis of early late (pp65), late (gB), and true late (pp28) genes is reduced significantly in viperin-expressing cells compared with control [52]. Because it interferes with the secretion of soluble proteins by disrupting lipid rafts of the plasma membrane, viperin likely exerts its antiviral effects by preventing virion assembly at a late stage of the viral life cycle.

An IFN-inducible family of proteins, previously known as the p200 family, has recently been demonstrated to suppress HCMV replication. This family, now designated PYHIN, comprises homologous human and mouse proteins that have an N-terminal Pyrin domain (PYD) and 1 or 2 partially conserved 200-residue C-terminal domains (HIN domain) [53]. These proteins are pleiotropic, based on their ability to bind to various target proteins (e.g., transcription factors, signaling proteins, and tumor suppressors) and modulate various cell functions. Increasing evidence implicates them as regulators of many processes, including proliferation, differentiation, apoptosis, senescence, inflammasome assembly, and the control of organ transplants.

Two members of the PYHIN family, AIM2, and IFN inducible protein 16 (IFI16), bind to and function as PRRs of virus-derived intracellular DNA [8]. In particular, IFI16 interacts with the adaptor molecule ASC and procaspase-1, forming a functional inflammasome during Kaposi sarcoma-associated herpesvirus (KSHV) infection [54]. Moreover, the induction of IRF3 and NF-*κ*B-dependent genes by herpes simplex virus (HSV)-1 infection in RAW264.7 cells is impaired by siRNA that targets p204, the murine ortholog of IFI16 [55].

Using two approaches, we recently determined IFI16 to be an antiviral factor against HCMV [56]; IFI16 expression was knocked down by specific siRNA, enhancing HCMV replication, and transduction with dominant-negative IFI16 (lacking the PYD) increased HCMV replication, whereas overexpression of wild-type IFI16 impaired HCMV viral yield. In the latter set of experiments, early (E) and late (L), but not IE, mRNA and protein were downregulated, indicating that IFI16 exerts its antiviral effects by hindering viral DNA synthesis. The HCMV UL54 (also called *pol*) is the catalytic subunit of HCMV DNA polymerase and represents a prototypical early gene required for viral DNA replication. We have shown that IFI16 overexpression induces a significant inhibition of UL44, UL54, and UL83 mRNAs. These data were also confirmed at protein level. Moreover, transfection and electrophoretic mobility shift assay experiments performed with nuclear extracts of HCMV infected cells demonstrated that the UL54 promoter is the target of IFI16-induced viral suppression. In fact, using luciferase constructs that were driven by a site specifically mutated HCMV DNA polymerase (UL54) promoter, we noted that IFI16 suppresses UL54 transcription [56]. These data indicate that IFI16 has antiviral activity against HCMV and provide novel insights into the functions of IFI16 as a viral restriction factor.

**Figure 2:**
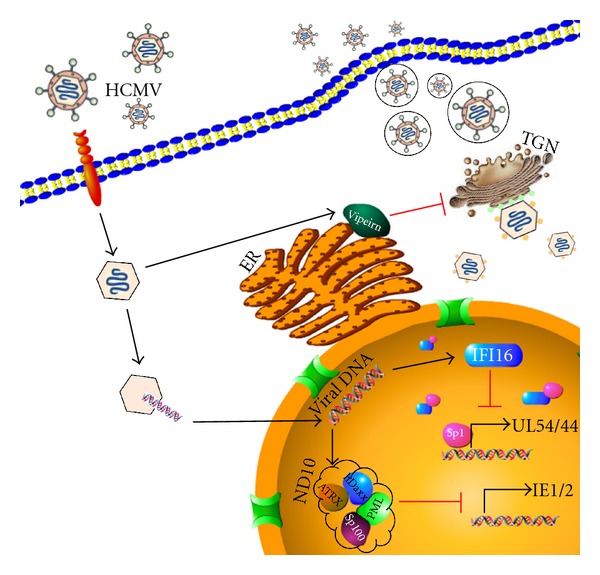
Type I IFN restriction factors that target HCMV. Type I interferons (IFN) are effector molecules of the immune response to virus. This antiviral action is mediated by IFN-stimulated genes. ND10 proteins are induced by IFN and function as part of an intrinsic antiviral defense mechanism of the cell by suppressing viral immediate early (IE) gene expression. The IFN-inducible protein IFI16 interacts with and displaces the transcription factor Sp1 from its DNA cognate element, the IR-1 element, in the viral UL54 promoter. This interaction inhibits the UL54 promoter and decreases HCMV DNA synthesis. The IFN-inducible protein viperin exerts its antiviral effects at a late stage of the HCMV life cycle. During infection, viperin is redistributed from the endoplasmic reticulum (ER) to the Golgi apparatus (TGN, *trans* Golgi network) and then to cytoplasmic vacuoles that contain gB and pp28.

Type I IFN-induced restriction factors, briefly described and summarized in Figure 2, constitute a potent antiviral defense mechanism against HCMV infection, rendering viral replication a true hurdle race. 

### 3.1. Strategies Adopted by HCMV to Escape Activity of IFN Restriction Factors

In response to the antiviral action of type I IFN factors, HCMV has evolved regulatory proteins and counteracting mechanisms that subvert and inactivate such factors. For example, IE1 disrupts ND10 by inducing the deSUMOylation of PML [47]. Recent evidence has demonstrated that HCMV relocates viperin from the endoplasmic reticulum to the mitochondria, where it reduces the generation of ATP, disrupting the actin cytoskeleton and enhancing viral infection [57]. Nuclear IFI16 appears to become inactivated, following its egress from the nucleus, during early gene expression by molecular mechanisms that appear to rely on protein ubiquitination (Landolfo et al. unpublished results). 

## 4. Function of Innate Immunity Cells during HCMV Infection

HCMV infects host cells of the myeloid lineage, such as monocytes, M*φ*s, and myeloid DCs. Despite their resistance to HCMV infection, lymphoid lineage cells, such as NK cells and PDCs [58], are also activated rapidly by viral components, confirming the importance of early virus-host interactions in the induction of prompt host defense mechanisms. However, HCMV has developed myriad immunoevasion strategies, allowing it to subvert host cell functions for its own advantage.

### 4.1. HCMV Efficiently Infects APCs and Employs These Cells as Vehicle of Viral Dissemination

APCs, including monocytes and various DC and M*φ* subsets, are critical in initiating specific naive and memory T-cell responses and coordinating and modulating host responses. Nevertheless, it is evident that HCMV hijacks these cells, transforming them into vehicles for viral dissemination in the first phase of infection and sheltered reservoirs in which the virus can persist, reactivate, and replicate under favorable conditions [59].

HCMV infects myeloid APCs, based on the detection of viral genome and antigens [60–63]. Monocytes do not support productive viral replication, and viral gene expression is restricted to early events [64, 65], whereas infected fully differentiated M*φ*s and myeloid DCs undergo lytic viral cycles, express late HCMV genes, release infectious virus, and stimulate T-cell responses *in vitro* [62, 63, 66, 67]. Thus, the ability of HCMV to replicate in myeloid cells depends on their stage of differentiation, as shown in an experimental model of HCMV latency, which was established by infecting human monocytes with a clinical isolate *in vitro*, in which monocytic differentiation to M*φ*s or DCs induced viral reactivation [68].

**Figure 3:**
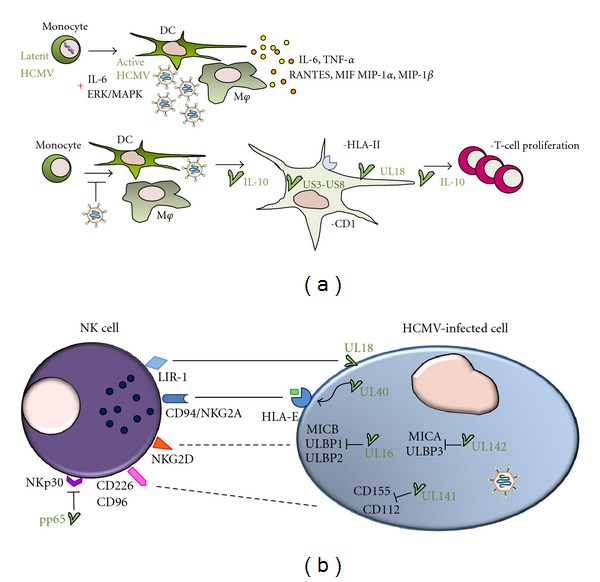
Cells of innate immunity, activation and virus counterattack. (a) HCMV reactivates from latency in infected monocytes by inflammation or cellular differentiation, in which IL-6 and ERK/MAPK signaling are involved. Differentiated macrophages (M*φ*) and dendritic cells (DC) are permissive for viral replication and, once infected, release proinflammatory factors. HCMV hampers the ability of M*φ* and DC to properly differentiate from monocytes and present antigens to T lymphocytes by downregulating surface expression of CD1 and HLA class II molecules. DC-induced T-cell proliferation also decreases through mechanisms that involve virally encoded IL-10 and pUL18. IL-6, interleukin-6; TNF-*α*, tumor necrosis factor-*α*; MIF, macrophage migration inhibitory factor; MIP-1*α*, macrophage inflammatory protein-1*α*; MIP-1*β*, macrophage inflammatory protein-1*β*. +; upregulation or activation, −; downmodulation or inhibition. (b) HCMV-encoded proteins modulate NK-cell recognition of infected cells. pUL40 binds to HLA-E and upregulates its surface expression, potentiating its interaction with the inhibitory receptor CD94/NKG2A. pUL18, an HLA-I viral homolog, binds to the inhibitory receptor LIR-1. Expression of the ligands of the activating receptor NKG2D is inhibited by pUL16 (which targets MICB, ULBP1, and ULBP2) and pUL142 (targeting MICA and ULBP3). pUL141 prevents the expression of CD112 and CD155, ligands of the activating receptors CD226 and CD96, whereas pp65 interferes with the signal transduction of the activating receptor NKp30. Solid lines: possible interactions resulting in NK-cell inhibition. Dotted lines: impairment of interactions between activating receptors and their ligands.

During the differentiation of DC progenitors to mature DCs *ex vivo*, chromatin structure is altered, permitting robust IE expression and, consequently, reactivation of latent HCMV [69]. Consistent with these observations, the inhibition of viral lytic genes that occurs during latency in undifferentiated myeloid precursors, including monocytes, is attributed to their inability to sustain high IE levels; the histone modifications present on the MIEP impart on it a repressive chromatin structure preventing transcriptional activity [70]. Recent evidence implicates IL-6 signaling and activation of the ERK/MAPK pathway in HCMV reactivation from potentially permissive cells, such as interstitial DCs [71]. Thus, myeloid cell differentiation, which is driven by inflammation and proinflammatory factors, such as IL-6, contribute to reactivation of latent HCMV infection (Figure 3(a)).

Conversely, the virus can enhance inflammation by acting on APCs; HCMV infection of peripheral monocytes induces a proinflammatory state, resulting in their adhesion to endothelial cells and transendothelial migration [72] and the secretion of proinflammatory cytokines and chemotactic factors [73]. Further, Mo-DCs [74, 75] and monocyte-derived M*φ*s [76] release proinflammatory factors on productive HCMV infection *in vitro*.

### 4.2. Immunoevasion Mechanisms Adopted by HCMV against APC Responses

In addition to enhancing inflammation for its own sake, HCMV hampers APCs in taking up and presenting the proper antigen to T lymphocytes. Several counteracting mechanisms have been evolved by HCMV to circumvent APC activity (Figure 3(a)). Immunoevasive viral transcripts, such as gpUS3 and gpUS8, that block human leukocyte antigen (HLA-) mediated antigen presentation pathways predominate during the early phases of HCMV infection of myeloid DCs [77]. HCMV inhibits the differentiation of M*φ*s and DCs from monocytic precursors, blocking their phagocytic, migratory, and allostimulatory activities [78, 79].

HCMV also impairs the immunophenotype and function of differentiated APCs. For example, it downmodulates integrin-like receptors, such as CD11b/CD18 (CR3) and CD11c/CD18 (CR4), on the surface of monocyte-derived M*φ*s, reduces their phagocytic activity [80], and impairs migration by downregulating CCR1 and CCR5, reorganizing the cytoskeleton, and inducing the secretion of soluble inhibitors [76]. Further, HCMV-infected, immature Mo-DCs have fewer surface HLA class I and class II molecules and impaired migratory and immunostimulatory capacity [74, 81, 82]. The virus also inhibits Mo-DC maturation and impedes the migration of mature DCs in response to lymphoid stimuli and induction of T-cell proliferation [75, 82, 83]. Similarly, on infection with HCMV, activation markers are downregulated in mature Langerhans DCs, decreasing their ability to stimulate T-cell proliferation [84, 85].

Many events have been implicated in the HCMV-induced impairments to immunostimulation by DCs, such as the release of soluble CD83 [86], upregulation of apoptosis-stimulating molecules [87], expression of the HCMV-encoded HLA class I-like homolog pUL18 [88], and secretion of the viral homolog of IL-10, which is expressed during the productive phase of infection (cmvIL-10) [89]. cmvIL-10 also impairs CD1-mediated antigen presentation (by reducing CD1 transcription) [90], monocyte function [91, 92], and TLR-induced transcriptional activation of IFN *α*/*β* genes in PDCs [93]. cmvIL-10 enhances HCMV infectivity by upregulating the viral entry receptor DC-SIGN [89]. Thus, secretion of cmvIL-10 during HCMV infection has many effects in hindering APC function.

### 4.3. APCs and HCMV: A Double-Edged Sword

Despite the subversion of APC function by the virus, specific effector and memory T cells develop during acute HCMV infection [94, 95] and robust adaptive immune responses develop to many HCMV antigens, of which IE1 is a significant target of CD4^+^ and CD8^+^ T-cell responses [94]. Whereas immunostimulation by DCs is profoundly impaired by the virus, HCMV-infected M*φ*s induce efficient T-cell activation through presentation of endogenous IE antigen [62]. Further, mechanisms of crosspresentation, the exogenous acquisition of antigen that is presented directly to CD8^+^ T cells without endogenous processing, are also initiated during HCMV infection of APCs [96]. However, the effective role of cross-presentation in inducing an efficient cellular imunity to HCMV has not yet been addressed.

### 4.4. NK Cell Activation during HCMV Infection

NK cells are a critical component of early innate immune responses against certain viruses, including HCMV. Individuals with NK-cell defects have increased susceptibility to herpesviruses and, in particular, HCMV [97, 98]. Moreover, the extensive mechanisms that HCMV implements to prevent NK-cell activation are indirect evidence of their importance in the innate response to HCMV.

NK cells accumulate rapidly in several organs during viral infections, taking active part in the direct elimination of injured target cells by cytotoxicity and in the activation and recruitment of other cells of the immune system by secreting cytokines and chemokines, including IFN-*γ* and TNF-*α* [99]. In secondary lymphoid organs and damaged tissues, NK cells establish a dialog with APCs, thus regulating innate and adaptive immune responses [100].

NK cells recognize virus-infected cells, using a repertoire of stimulatory and inhibitory cell surface receptors [101] that control NK-cell activation, proliferation, and effector functions; their cytotoxic function depends primarily on stimulatory receptors. Different receptors are expressed to respond to different ligands on target cells: (i) HLA class I molecules (HLA-I), frequently downmodulated in virus-infected cells are recognized by specific inhibitory receptors, including killer cell-Ig-like receptors (KIRs), leukocyte Ig-like receptor 1 LILRB1 (LIR-1), and C-type lectin receptor CD94/NKG2A; (ii) pathogen-derived molecules are recognized by activating receptors, and (iii) self-proteins that are upregulated on “stressed” or damaged cells bind to a major activating receptor, NKG2D [102].

### 4.5. Mechanisms of Viral Immunoevasion Employed against NK Cells

Many inhibitory receptors on NK cells, including KIRs and LIR-1, recognize HLA-I, and under normal conditions, the engagement of inhibitory receptors by self-molecules suppresses NK-cell attack. However, HCMV is able to reduce cell surface expression of HLA-I by several mechanisms (reviewed in [103]). Consequently, it was predicted that according to the *missing self* hypothesis, low levels of HLA-I on HCMV-infected cells render them vulnerable to NK-cell lysis [104]. Yet, NK cells fail to discriminate between normal and infected cells on the basis of virus-induced HLA-I downmodulation [105, 106]. HCMV circumvents other aspects of the NK cell-target cell interaction [107], and HCMV-infected cells become resistant to be attacked by NK cells, due to a vast array of virally encoded immunomodulatory molecules [108].

Two mechanisms describing HCMV-mediated inhibitory signalling have been proposed. In the first, HCMV encodes for pUL18, an HLA-I homolog [109] that, like HLA-I, binds *β*2-microglobulin [110] and peptides [111] and engages the inhibitory receptor LIR-1 with 1000-fold higher affinity compared with HLA-I [112–114]. pUL18 inhibits LIR-1^+^ NK cells but has additional effects, because LIR-1 is expressed on other cells of the immune system, including APCs [115]. For example, the binding of pUL18 to DCs impairs cell migration and CD40 ligand-induced maturation, reducing T-cell proliferation [88]. Thus, pUL18 can be exploited by HCMV to avoid host immune responses [116]. Clinical isolates of HCMV retain *UL18*, underscoring its importance for viral survival in the host [117, 118].

In the second mechanism, HCMV uses the host HLA-E pathway to suppress NK cells through the inhibitory receptor complex CD94/NKG2A. A nonameric peptide that is derived from the leader sequence of the viral protein pUL40 is a canonical ligand for the nonclassical HLA-I molecule HLA-E and promotes HLA-E expression on the cell surface [119–121], facilitating the interaction between HLA-E and CD94/NKG2A receptor and conferring resistance to NK-cell lysis [122–125].

Because the decision by NK cells to attack relies on the sum of signals from inhibitory and activating receptors, it is important for the virus to prevent the engagement of activating receptors. HCMV encodes five genes that impede signaling by activating receptors on NK cells: UL16, UL141, UL142, UL83, and microRNA-UL112-1 (miRNA-UL112) [108]. pUL16, pUL142, and miRNA-UL112 inhibit the expression of ligands of a major activating receptor, NKG2D. In humans, the ligands for NKG2D are the human major histocompatibility complex (MHC) class I chain-related genes (MIC)A, MICB, and ULBP1-6 molecules, which are particularly expressed under stress and on stimulation by innate cytokines that are produced during viral infections (reviewed in [126]).

Because NKG2D has an important role in controlling both NK- and T-cell-mediated immunity, it is reasonable that this receptor and its ligands forced the virus to evolve specific strategies of evasion. pUL16 prevents cell surface expression of MICB, ULBP1, and ULBP2 by binding and sequestering them in the endoplasmic reticulum or Golgi [127–129]. The selective pressure that is exerted by pUL16 likely contributes to drive the diversification of NKG2D ligands, eventually leading to the emergence of proteins that do not interact with UL16, such as MICA and ULBP3; the expression of which, however, is countered by the HCMV protein pUL142, which retains them in the *cis*-Golgi [130–132]. In addition, MICB is under the control of the virally encoded miRNA-UL112 which specifically reduces its cell surface expression [133].

Another tactic that was evolved by HCMV to interfere with activating receptors relies on pUL141, which sequesters the adhesion molecules CD155 (PVR/necl-5) [134] and CD112 (nectin-2) intracellularly [135]; these proteins are ligands for the NK-cell activating receptors CD226 (DNAM-1) and CD96 (TACTILE) [136]. Notably, pUL141 is the most robust modulator of NK cells that has been tested *in vitro*, inhibiting a wide range of human NK-cell populations [134]. This important function explains in part the increased resistance to NK-cell lysis of low-passaged HCMV clinical isolates compared with the laboratory strain AD169 [105], from which 13–15 kbp of DNA has been deleted due to extensive passaging *in vitro* [137], a segment that contains UL141 [108, 134].

The pp65 tegument protein also affects NK-cell functions, dissociating the *ζ*-chain from the natural cytotoxicity receptor NKp30 and preventing it from transducing signals through an unknown mechanism [138]. The outcome of these disparate strategies is impaired NK-cell-mediated recognition and elimination of HCMV-infected cells, as depicted in Figure 3(b).

### 4.6. NK Cells and HCMV: Windows of Opportunity for Host Counterattack

Despite the many viral strategies that modulate the antiviral functions of NK cells, there is a *window of opportunity *during which host responses can prevail, potentially rendering infected cells detectable by the immune system. Such a circumstance could be achieved through several mechanisms, depending on genetic variations in the virus and host. For example, some allelic variants of NKG2D ligands are unaffected by known viral strategies. The MICA*008 allele, the most frequent allele in several populations, does not bind to viral pUL142. This variant has a truncated cytoplasmic tail, making it resistant to pUL142 and allowing it to persist on the surface of infected cells, where it can induce NK cells to lyse [132, 139]. This finding suggests that UL142 may be driving the selection of certain MICA alleles in humans [140, 141].

Genetic variations have also been detected in *UL142* from different clinical isolates of HCMV, some of which are more efficient in downregulating MICA expression [132]. Variations have also been identified in pUL40 and pUL18 [117, 118, 124].

Despite of the wide range of strategies that are used by HCMV to modulate NK-cell function, there is still the possibility of a time interval during which host responses prevail. MICA and MICB expression appears to be regulated by IE1 and IE2 proteins, indicating that viral *trans *activation is largely mediated by these HCMV gene products [142]. Notably, this effect might allow NK-activating ligands to be expressed before late immunoevasion genes are expressed and exert their effects. Collectively, this evidence suggests that the cellular response to infection could be sufficiently robust in some individuals against certain viral strains and/or at a specific time after infection, allowing to achieve elevated, functionally relevant levels of activating signals.

### 4.7. Interplay between NK Cells and APCs during HCMV Infection

NK-DC crosstalk is bidirectional, NK cells can kill immature DCs or promote their maturation, and in turn, mature DCs can stimulate NK-cell cytotoxicity and proliferation. These processes depend primarily on the activating receptors NKp30 and DNAM-1 and on the production of cytokines, such as IL-12, IL-15, IL-18, and IFN-*α*/*β* [100, 143–149].

Recent evidence has demonstrated that NK cells regulate HCMV infection through interactions with autologous APCs, such as Mo-DCs and polarized M*φ*s; NK cells respond vigorously against infected Mo-DCs by producing IFN-*γ* and becoming cytotoxic, where NKp46 and DNAM-1 have a dominant role [150]. Such a response is evident early after infection, whereas later, the virus-mediated downregulation of the DNAM-1 ligands CD155 and CD112 prevails, illustrating the significance of the course of infection with regard to the efficacy of the host response. Further, the production of IFN-*γ* by NK cells is influenced by the polarization of M*φ*s, wherein proinflammatory M*φ*s induce more efficient IFN-*γ* responses than anti-inflammatory M*φ*s on HCMV infection [151]. 

## 5. Early Events of HCMV Replication as Potential Targets for Therapeutic Intervention

The identification of cellular and viral components that regulate early HCMV-host cell interactions has increased our understanding of the pathogenesis of HCMV diseases and formed the rationale for the design of novel antiviral interventions that target these initial events.

The need for anti-HCMV drugs with novel mechanisms of action is underscored by the findings that conventional standard therapy is often associated with considerable adverse events and that prolonged treatment can lead to the emergence of drug-resistant strains [152]. Further, agents that target viral DNA polymerase are unable to prevent viral attachment or entry or the expression of IE proteins, which mediate proinflammatory responses and immunomodulation. Thus, blocking pre-IE events and IE expression and function may represent an alternative strategy of combating HCMV-induced immunopathological phenomena [153]. Several molecules that effect such outcomes have been identified (reviewed in [154]). However, with the sole exception of hyperimmune globulin preparations, compounds that target HCMV attachment and entry remain at the preclinical stage of development. We briefly review the properties of those experimental agents that have been shown to inhibit HCMV attachment and entry *in vitro*.

The adsorption of HCMV virions to cell surface heparan sulfate proteoglycans (HSPGs) is mediated by positively charged regions of the viral gM/gN complex and is essential for stabilizing virions at the cell surface prior to the engagement of entry receptors [4]. Several experimental inhibitors of HCMV attachment have been characterized, including sulfated polysaccharides, lactoferrin, and peptide-derivatized dendrimers. Negatively charged polyanions, such as sulfated polysaccharides from bacteria, algae, and animals and semisynthetic compounds, such as dextran sulfate and pentosan polysulfate, disrupt the electrostatic interactions between the positively charged region of HCMV envelope glycoproteins and the negatively charged sulfate/carboxyl groups of heparan sulfate (HS) chains in HSPGs; these compounds show potent anti-HCMV activity against laboratory strains and clinical isolates [155]. HSPGs can also be bound by the N-terminal region of lactoferrin, an iron-binding glycoprotein that exists in most mucosal secretions and body fluids, suggesting that it acts by preventing virions from tethering to the cell surface [156].

 Dendrimers are synthetic hyperbranched molecules that may have potential applications as antivirals, based on their small size (nanomolar), ease of preparation, and ability to display multiple copies of surface groups (multivalency) that are required for recognition, including the initial interactions that occur between an infecting virus and the target cell [157]. Recently, two peptide-derivatized dendrimers, SB105 and SB105_A10, were shown to inhibit HCMV replication directly by preventing viral adsorption to HSPGs onto cells [158, 159].

The use of compounds that target viral attachment could be curbed by the cell-to-cell spread of clinical HCMV isolates. In a normal host, however, the release of cell-free virus depends on the site of infection; whereas cell-free viral transmission during hematogenous dissemination is believed to be unlikely (because HCMV replication is highly cell associated), cell-free virus is commonly found in body fluids, such as urine, saliva, and breast milk, often at high titers [160]. Thus, molecules that block viral adsorption may be used to prevent HCMV transmission *via* such excretions.

 HCMV-exploits its coding capacity for glycoproteins to form different envelope complexes [3]. The gH/gL heterodimer can participate in two distinct glycoprotein complexes; it can associate with gO to form a heterotrimeric complex that regulates pH-independent fusion at the cell surface in fibroblasts or it associates with pUL128, pUL130, and pUL131 to form a pentameric complex, required for entry by endocytosis, followed by low pH-dependent fusion in endothelial and epithelial cells, DCs, and monocytes [67, 161–163]. gB is also required for viral entry and cell-to-cell spread [164]. Thus, compounds that bind to virion components that mediate entry or interfere with the protein-protein interactions required to induce membrane fusion can be termed HCMV entry inhibitors.

Experimental agents that have been shown to interfere with HCMV entry include CFI02, *β*-peptides, and CpG ODNs. gB is the target of a small-molecule thiourea derivative, CFI02, which suppresses HCMV replication. Mechanism-of-action studies indicate that CFI02 acts at an early stage in HCMV replication by inhibiting gB-mediated fusion of the virion envelope to the cell membrane [165]. Further, heptad repeat motifs, characteristic of *α*-helical coiled-coil interactions, have been identified within gB and gH. Peptides that correspond to these regions have been shown to inhibit the entry of clinical and laboratory HCMV strains, thus providing the proof of concept that blocking the coiled-coil interactions required for viral entry is a feasible strategy of preventing HCMV infection [166]. These potential new targets for therapeutic intervention have been exploited, based on the development of oligomers of *β*-aminoacids (*β*-peptides) that mimic the heptad repeat domain of gB and block viral infection during virus-cell membrane fusion [167]. *β*-peptides showed to be more potent than gB-derived *α*-peptides and blocked the activation of the type I IFN pathway in HCMV-infected fibroblasts [21], suggesting that *β*-peptides can impede both HCMV replication and viral-induced immunopathogenesis.

 Short synthetic oligodeoxynucleotides that contain deoxycytidyl-deoxyguanosine motifs (CpG ODNs) can mimic bacterial and viral DNA to stimulate TLR9 and activate innate responses [168, 169]. Their antiviral activity has been proposed to be secondary to CpG-induced IFN responses that are triggered through TLR9 activation. Luganini et al. [170] recently reported, however, that *in vitro* replication of HCMV was suppressed by several CpG ODNs in a TLR9-independent mechanism. The B-class prototype CpG ODN 2006 was shown to prevent the nuclear localization of pp65 and input viral DNA, thus suggesting that it inhibits HCMV entry [170]. Notably, when added after the onset of HCMV replication, CpG ODN 2006 stimulates viral replication [31], as discussed, indicating that once the virus establishes its transcriptional programs, it takes advantage of the TLR9 stimulation pathway to propagate. These findings also suggest that CpG ODNs should be considered for antiviral intervention solely to prevent HCMV infection.

Yet, the window of opportunity for the mentioned experimental compounds that target the attachment and entry phases of HCMV infection is narrow. Their development as candidate drugs for future intervention should be considered in combination with conventional anti-HCMV therapeutics, such as ganciclovir and foscarnet that inhibit viral replication.

 Conversely, intravenous immunoglobulins that are enriched for antibodies against HCMV (HCMV-IVIG) have been approved for use in preventing HCMV diseases in transplant recipients. The rationale for their clinical application lies in their ability to neutralize the virus and prevent entry into several cell types. Therefore, HCMV-IVIG represents the first example of a drug capable of blocking a pre-IE event that has been extensively used in patients at risk of HCMV disease. Further, the immunomodulatory activity of IVIG [171] might help reduce HCMV-induced immunopathology. However, in spite of their widespread clinical application, the role of HCMV-IVIG in the prevention of HCMV infection and disease remain to be fully elucidated. In fact, prophylactic administration of HCMV-IVIG has been associated with improved total survival, reduced HCMV disease, and lower HCMV-associated deaths in solid organ transplant recipients [172], whereas in patients who are undergoing hematopoietic stem cell transplantation, routine prophylaxis with HCMV-IVIG remains controversial [173]. Moreover, observational clinical studies indicate that administration of HCMV-IVIG to pregnant woman with primary HCMV infection may be effective in treating and preventing fetal infection [174].

The low neutralization potency of these preparations, however, may limit their clinical use. Thus, human monoclonal antibodies (mAbs) that neutralize HCMV infection have recently garnered interest as more effective and safer passive immunotherapeutic agents. Panels of human mAbs against gB and gH [175] or those that recognize conformational epitopes that require two or more proteins of the gH/gL/pUL128-131 pentameric complex [176] were developed from immortalized memory B cells of HCMV-immune donors. Notably, the human mAbs against the UL128-131 locus gene products [161] showed a neutralizing activity 2-3 logs more potent than neutralizing mAbs directed to gB or gH [176]. Although their protective activity *in vivo* remains to be investigated, these new human mAbs are promising next-generation immunotherapeutic compounds for the therapy/prophylaxis of HCMV infection and disease.

## 6. Concluding Remarks

The complex interaction between HCMV and the host begins immediately on viral contact with many cell types, including innate immune cells. Virion recognition and binding and entry-related events induce inflammation and IFN responses, the latter upregulating restriction factors that, in turn, contribute to the creation of an intracellular antiviral state. However, the induction of the IFN response is modulated by many counteracting viral mechanisms, as well as the inactivation of IFN restriction factors and modulation of innate cell functions that facilitate evasion of host intrinsic and innate immunity.

The identification of the mechanisms of host-HCMV interactions during attachment and entry has provided the rationale for the design of novel experimental compounds that target these events. Blocking the early phases of infection may provide a window of opportunity that allows such interventions to inhibit HCMV gene expression and replication and modulate inflammatory and IFN host responses, thus hindering viral-induced immunopathogenesis.

 HCMV uses several immunoevasion strategies to evade host NK cells and APCs, most of which involve protein products of L viral genes that are used to complete the viral cycle. Novel therapeutics that block the viral cycle before the late stages of replication might also prevent HCMV from exploiting such strategies, thus increasing the immunocompetence of the host.
